# Supplementary material for: Prevalence of DSM-5 mild and major neurocognitive disorder in India: Results from the LASI-DAD
Source: PLoS One. 2024 Feb 7;19(2):e0297220. doi: 10.1371/journal.pone.0297220 (PMC10849236; doi:10.1371/journal.pone.0297220)
Supplement: S1 Table — (DOCX) [file pone.0297220.s001.docx]

Supplemental Table 1. Prevalence of major and mild neurocognitive disorder by demographic characteristics using orientation as a cognitive domain to define neurocognitive disorder: Results from LASI-DAD (N=4096)

| Characteristic | Sample size (unweighted) | Mild NCD, weighted % | Major NCD, weighted % |
| --- | --- | --- | --- |
| Full sample | 4096 | 18.6 | 7.8 |
| Age |  |  |  |
| 60-64 | 1290 | 16.3 | 4.2 |
| 65-69 | 1179 | 17.7 | 7.2 |
| 70-79 | 720 | 20.1 | 9.5 |
| 80+ | 453 | 24.5 | 16.2 |
| Education |  |  |  |
| No formal education | 2009 | 20.2 | 11.7 |
| Secondary education or less | 1076 | 18.8 | 5.5 |
| Tertiary education or higher | 1010 | 16.0 | 4.0 |
| Sex |  |  |  |
| Male | 1889 | 17.8 | 8.8 |
| Female | 2207 | 19.5 | 6.8 |
| Literacy |  |  |  |
| Literate | 1777 | 18.8 | 5.2 |
| Illiterate | 2319 | 18.5 | 10.2 |
| Residential status |  |  |  |
| Urban | 1556 | 16.7 | 5.2 |
| Rural | 2539 | 21.1 | 11.1 |

Legend. Raw sample sizes are provided with weighted proportions. This table differs from Table 3 in the manuscript in that the algorithmic definition of neurocognitive disorder included a fifth domain of orientation as a domain.
