# Supplementary material for: Prevalence of DSM-5 mild and major neurocognitive disorder in India: Results from the LASI-DAD
Source: PLoS One. 2024 Feb 7;19(2):e0297220. doi: 10.1371/journal.pone.0297220 (PMC10849236; doi:10.1371/journal.pone.0297220)
Supplement: S2 Table — (DOCX) [file pone.0297220.s002.docx]

Supplemental Table 2. Cross-tabulation of CDR scores with DSM-5 algorithmic neurocognitive disorder using orientation as a domain to define neurocognitive disorder: Results from LASI-DAD (N=2390)

|  | Algorithmic neurocognitive disorder, n | | |
| --- | --- | --- | --- |
| CDR score | Cognitively normal | Mild NCD | Major NCD |
| 0 | 715 | 44 | 5 |
| 0.5 | 975 | 341 | 175 |
| 1 | 21 | 16 | 70 |
| 2 | 2 | 0 | 21 |
| 3 | 0 | 0 | 5 |

Legend. Values reflect raw numbers of people in each cell defined by CDR score and algorithmic neurocognitive disorder.
