# Supplementary material for: Prevalence of DSM-5 mild and major neurocognitive disorder in India: Results from the LASI-DAD
Source: PLoS One. 2024 Feb 7;19(2):e0297220. doi: 10.1371/journal.pone.0297220 (PMC10849236; doi:10.1371/journal.pone.0297220)
Supplement: Attachment — Submitted filename: Responses1 to PLOSONE 20230930.docx [file pone.0297220.s003.docx]

October 7, 2023

Dear Editor,

We are pleased to submit a revision of PONE-D-23-26576 ("Prevalence of DSM-5 mild and major neurocognitive disorder in India: Results from the LASI-DAD"). We appreciate the feedback about the manuscript provided by the two Reviewers. We have internalized the excellent critiques and used them to revise the manuscript, including the Introduction, Methods, Results, and Discussion, to describe our study’s rationale and details around the methods more clearly. All revisions were guided by the Reviewers. Page and line numbers listed in our responses in this revision letter refer to locations in the revised manuscript and not the original manuscript. Text revisions in this letter and in the revised manuscript are highlighted in red.

**EDITOR COMMENTS**

**EDITOR COMMENT #1:** Please ensure that your manuscript meets PLOS ONE's style requirements, including those for file naming. The PLOS ONE style templates can be found at 
<https://journals.plos.org/plosone/s/file?id=wjVg/PLOSOne_formatting_sample_main_body.pdf> and 
<https://journals.plos.org/plosone/s/file?id=ba62/PLOSOne_formatting_sample_title_authors_affiliations.pdf>

**RESPONSE 1:** We confirm that the title page is formatted according to PLOS ONE style. We checked, and confirm that file naming conventions correspond to PLOS ONE requirements.

**EDITOR COMMENT #2:** Please include a copy of Table 5 which you refer to in your text on page 11 and 12.

**RESPONSE 2**: We apologize for the omission of Table 5 in our original submission. We inserted it in the text on page 13.

**EDITOR COMMENT #3:** Please upload a copy of Supporting Information Figure/Table/etc. Supplemental table 1 and 2 which you refer to in your text on page 12.

**RESPONSE 3**: We apologize for the omission of these Supplemental Tables in our original submission. We added them to a file “S1 Appendix.docx.”

**EDITOR COMMENT #4:** Please include captions for your Supporting Information files at the end of your manuscript, and update any in-text citations to match accordingly. Please see our Supporting Information guidelines for more information: <http://journals.plos.org/plosone/s/supporting-information>.

**RESPONSE 4**: We include names of the two Supplemental tables at the end of the manuscript, and uploaded the tables in a separate Word file, per the website provided.

**EDITOR COMMENT #5:** Please review your reference list to ensure that it is complete and correct. If you have cited papers that have been retracted, please include the rationale for doing so in the manuscript text, or remove these references and replace them with relevant current references. Any changes to the reference list should be mentioned in the rebuttal letter that accompanies your revised manuscript. If you need to cite a retracted article, indicate the article’s retracted status in the References list and also include a citation and full reference for the retraction notice.

**RESPONSE 5**: We have reviewed the reference list and believe it is complete and correct. We do not cite any retracted papers. We have changed the reference list to accommodate responses to Reviewers. We note those additions in responses to Reviewers 1 and 2, below.

**REVIEWER 1 COMMENTS**

**REVIEWER 1, COMMENT #1:** Equating neurocognitive disorder as constructed here with MCI and AD may have some issues. What implications do the sensitivity and specificity of the diagnostic construct used here have on the final prevalence estimates?

**RESPONSE 1**: We are grateful to the Reviewer for this critique. Since we refer to MCI only colloquially in two places in this manuscript, we replaced those instances with mild neurocognitive disorder so as not to confuse these related but different concepts.

**Introduction (page 4, lines 74-75):** “The present study aims to describe the prevalence of mild and major neurocognitive disorder in a nationally representative sample in India.”

**Discussion (page 14, lines 294-296):** “Especially given recent therapeutic advances in treatments for Alzheimer’s disease, in coming years there may be increased emphasis on defining and identifying those with preclinical dementia and milder impairments (e.g., mild neurocognitive disorder) (31).”

Additionally, we added a brief explanation of some differences between previous 10/66 criteria and DSM-5 criteria in the **Introduction (page 3, lines 65-73):** “There are several notable distinctions between 10/66 criteria, based on DSM-IV, and DSM-5. First, the former required impairment in memory and another cognitive domain, while the latter is more inclusive in the sense that it required cognitive impairment in any domain, not just memory. Second, DSM-IV criteria required evidence that cognitive deficits led to impaired everyday functioning that represents a significant decline from a previous level. The DSM-5 revised this requirement by requiring that cognitive deficits interfere with independence in everyday activities. The requirement in DSM-IV of a decline in everyday function from a prior level was difficult to assess in older adults in India for whom assessment of independent activities of daily living can be challenging.”

**REVIEWER 1, COMMENT #2**: What implications do the sensitivity and specificity of the diagnostic construct used here have on the final prevalence estimates?

**RESPONSE 2**: We thank the Reviewer for this thoughtful question. In response, we added the following to the **Results (page 11, lines 243-250):** “The high specificity of the DSM-5 classification indicates there are few false positives which would typically falsely inflate the prevalence. Thus, the reported prevalence is unlikely to be an overestimate insofar as clinical dementia ratings can be accepted as a gold standard. The sensitivity of the DSM-5 classification is 0.66, indicating a sizable number of people with dementia via the CDR but without major neurocognitive disorder via DSM-5; of these N=45 participants, 20 (44%) were classified with mild neurocognitive disorder and the rest (N=25) were mildly impaired based on informant criteria but had minimal to no evidence of objective cognitive impairment (Table 1).”

**REVIEWER 1, COMMENT #3**: The discussion can include something brief on the sensitivity analysis - why was it considered and what are the implications of the result of the sensitivity analysis?

**RESPONSE 2**: We added the following paragraph to the Discussion to provide an explanation for this sensitivity analysis **(Discussion, page 15, line 325 to page 16, line 333):** “Because orientation reflects an assessment of a person's general understanding of surroundings rather than the ability to remember, think, or attend to stimuli, orientation is not a domain listed in DSM-5. We kept it out of our algorithm for this reason and also to not contaminate the validation of our DSM-5 algorithm against CDR ratings. Orientation items were available to clinicians who rated CDRs using the online adjudication platform. That said, algorithms that include criteria for impairment in 1+ domains or 2+ domains such as ours, the total number of possible domains matters. We used four domains (memory, executive functioning, language, and visuospatial ability), while another algorithm using HCAP data from the US used those domains as well as orientation (26).”

**REVIEWER 1, COMMENT #3**: The article is silent on the linguistic diversity of India. How many language versions were used? Would linguistic diversity affect the estimates or not?

**RESPONSE 3**: We thank the Reviewer for pointing this out. In response, we have revised the manuscript in two ways. First, we added descriptive information about languages of test administration to **Table 1 (page 5).** Second, we added the following to the **Methods section (page 4, lines 99-107):** “LASI-DAD participants were interviewed in one of 13 languages; aside from Hindi and English, languages of administration corresponded to one's state of residence (Table 1). Cognitive tests and interview materials were carefully translated, back-translated, and rechecked for accuracy by field staff and study investigators. A previous study tested for differences in the correlational structure of the cognitive test battery by language of administration by testing configural, metric, and scalar measurement invariance, using available data on N=3,224 participants representing 11 of the 13 languages (interviews from Gujarat and Punjab had not yet been released at that time). There was minimal evidence of measurement differences across language groups.”

**REVIEWER 2 COMMENTS**

**REVIEWER 2, COMMENT #1:** Number of women in India reporting to dementia clinics/memory clinics is significantly less compared to men; and this gender bias has been reported widely. Please clarify this based on your data.

**RESPONSE 1**: We thank the Reviewer for this astute suggestion. We did not find a sex difference that other studies have found, however other studies have also reported data suggesting no sex difference in the prevalence of dementia in India. We have added a paragraph discussing this to the **Discussion (page 15, lines 314-324):** “We did not find a strong sex difference in the prevalence of neurocognitive disorder. Among women, the prevalence of mild neurocognitive disorder was slightly greater while the prevalence of major neurocognitive disorder was slightly lower, but not substantially so. While this pattern of results is consistent with several recent studies of dementia prevalence in India (35-38), other studies report higher dementia prevalence among women (15) and especially among women in rural areas (12), possibly due to higher risk factor profiles such as lower education, illiteracy, greater exposure to indoor pollution from cooking, and vascular risk factors (39,40). Data from high-income countries in Europe and North America are mixed in this regard; a recent study suggested that higher prevalence estimates of dementia among women, may be attributable to longer life expectancy among women as well as selection effects related to clinical versus population-based samples (39,41).”

**REVIEWER 2, COMMENT #2:** Please specify clearly where and how the sample were collected.

**RESPONSE 2**: We agree this information is crucial to the present paper, and have added the following information to the **Methods (page 4, lines 81-92):** “Sample. The Longitudinal Aging Study in India (LASI) is a nationally representative sample of health, social supports, and economic security in N=72,262 adults in India aged 45 years and older, sampled from 36 states and union territories using multistage area probability methods (18). Part of the LASI core interview entailed a brief cognitive assessment; those unable to complete cognitive tests provided an informant interview to gauge their general mental status. The Harmonized Diagnostic Assessment of Dementia for the LASI (LASI-DAD) is a substudy comprising N=4,096 participants (19). The LASI-DAD study employed a stratified, random sampling design, recruiting participants from the main LASI study residing in 18 geographically and linguistically diverse states (Gujarat, Uttranchal, Jammu & Kashmir, Punjab, Haryana, Delhi, Rajasthan, Uttar Pradesh, Madhya Pradesh, Bihar, Assam, West Bengal, Orissa, Maharashtra, Karnataka, Kerala, Tamil Nadu, and Telangana), collectively representing 91% of the population (19).”

**REVIEWER 2, COMMENT #3:** Authors are concluding that the data reported in the study has higher prevalence than previously reported data; however, line 84 & 85 suggests that the participants and informants were interviewed both from home and hospital. Hence this claim looks superfluous.

**RESPONSE 3**: As noted in response 2 to Reviewer 2, the study selected respondents using a stratified, random sampling design. Participants were interviewed in a hospital setting if they lived near a participating hospital and preferred to be interviewed there rather than at home. We do not believe the location of interview should affect our results. To avoid confusion for readers, we have revised the manuscript to remove the sentence the Reviewer is referring to.
